# Supplementary material for: HIV-1 Protease as DNA Immunogen against Drug Resistance in HIV-1 Infection: DNA Immunization with Drug Resistant HIV-1 Protease Protects Mice from Challenge with Protease-Expressing Cells
Source: Cancers (Basel). 2022 Dec 30;15(1):238. doi: 10.3390/cancers15010238 (PMC9818955; doi:10.3390/cancers15010238)
Supplement: Supplementary file 1 [file cancers-15-00238-s001.zip › cancers-2038683-SI.pdf]

Supplementary materials

Supplementary Tables

Supplementary Table S1. List of plasmids used in the study

Purpose	Abbreviated plasmid name	Plasmid backbone (vector, reference)	Antibiotic resistance gene	Promoter	Cloned synthetic protease (PR) gene	Abbreviated name	Mutations in PR
Prokaryotic expression vectors	pET15b-PR_A	pET15b	AmpR	lacI	Consensus FSU_A PR	PR_A	none
	pET15b-PR_Aimut				Inactivated consensus FSU_A PR	PR_Ai	D25N
	pET15b-PR_A2mut				Drug resistant (DR) FSU-A PR	PR_A2mut	M46I, I54V
	pET15b-PR_Ai2mut				Inactivated drug resistant FSU-A PR	PR_Ai2mut	D25N; M46I, I54V
	pET15b-PR_A3mut				DR FSU-A PR	PR_A3mut	M46I, I54V, V82A
	pET15b-PR_Ai3mut				Inactivated DR FSU-A PR	PR_Ai3mut	D25N; M46I, I54V, V82A

Eukaryotic expression vectors	pVAX1	pVAX1 (Invitrogen)	KanR	IE CMV	none	none	none
	pKCMVPR_B (Hallengard et al., 2011)	pKCMV (Kjerrstrom and Wahren, 1999)			HIV-1 HXB-2 PR	PR_B	none
	pKCMVPR_Bi (Hallengard et al., 2011)	pKCMV (Kjerrstrom and Wahren, 1999)			Inactivated HIV-1 HXB-2 PR	PR_Bi	D25N
	pVax_PR_B	pVAX1			HIV-1 HXB-2 PR	PR_B	none
	pVax_PR_Bi				Inactivated HIV-1 HXB-2 PR	PR_Bi	D25N
	pVax_PR_A				Consensus FSU_A PR	PR_A	none
	pVax_PR_Ai				Inactivated consensus FSU_A PR	PR_Ai	D25N
	pVax_PR_A2mut				DR consensus FSU-A PR	PR_A2mut	M46I, I54V
	pVax_PR_Ai2mut				Inactivated DR consensus FSU-A PR	PR_Ai2mut	D25N; M46I, I54V
	pVax_PR_A3mut				DR consensus FSU-A PR	PR_A2mut	M46I, I54V, V82A

	pVax_PR_Ai3mut				Inactivated DR consensus FSU-A PR	PR_Ai3mut	D25N; M46I, I54V, V82A
Lentiviral vectors	pLVPR_A2mut	pRRLSIN.cPPT.PGK (Addgene, #12252)	AmpR	hPGK	DR consensus FSU-A PR	PR_A2mut	M46I, I54V
	pLVPR_Ai2mut				DR consensus FSU-A PR	PR_Ai2mut	D25N; M46I, I54V
	pLVPR_A3mut				DR consensus FSU-A PR	PR_A2mut	DM46I, I54V, V82A
	pLVPR_Ai3mut				Inactivated DR consensus FSU-A PR	PR_Ai3mut	D25N; M46I, I54V, V82A

Supplementary Table S2. Statistical analysis of the differences in the frequencies of CD8+ T cells producing cytokines in response to stimulation with protease derived peptides in pooled groups of PR_A immunized mice (PR_Ai, PR_Ai2mut and PR_Ai3mut) and mice receiving empty vector pVAX1 after booster immunization (Series II, Table 1). Percent of responding CD8+ T cells was assessed by flow cytometry with ICCS (see Materials and Methods for details). Sequences of peptides used for stimulation are presented in Fig. 1C. Statistical analysis was done using Mann-Whitney U-test, p values <0.05 were considered as significant (given in red).

Peptide	Cytokine produced by peptide stimulated CD8+ T cells	P value
A1-15	IFN-γ	0,0485
	IL-2	0,0485
	TNF-α	0,0162
	IFN-γ/ IL-2/ TNF-α	0,0465
A31-56	IFN-γ	0,7798
	IL-2	0,0990
	TNF-α	0,5495
	IFN-γ/ IL-2/ TNF-α	0,0687
A31-56dr	IFN-γ	0,7778
	IL-2	0,0040
	TNF-α	>0,9999
	IFN-γ/ IL-2/ TNF-α	0,7798
A56-70	IFN-γ	0,5455
	IL-2	0,3475
	TNF-α	0,0485
	IFN-γ/ IL-2/ TNF-α	0,7818
AB71-85	IFN-γ	0,0162
	IL-2	>0,9999
	TNF-α	0,7798
	IFN-γ/ IL-2/ TNF-α	0,0465

AB71-85dr	IFN-γ	0,7778
	IL-2	>0,9999
	TNF-α	0,7818
	IFN-γ/ IL-2/ TNF-α	0,5535
AB75-84	IFN-γ	0,0040
	IL-2	0,0162
	TNF-α	0,0040
	IFN-γ/ IL-2/ TNF-α	0,0040
A76-90	IFN-γ	0,2000
	IL-2	0,2020
	TNF-α	0,0465
	IFN-γ/ IL-2/ TNF-α	0,0040
A76-90dr	IFN-γ	0,7778
	IL-2	0,2000
	TNF-α	0,3475
	IFN-γ/ IL-2/ TNF-α	0,3475

Supplementary Table S3. Statistical analysis of differences in the frequencies of CD8+ T cells producing cytokines in response to stimulation with protease derived peptides between groups of mice DNA immunized with PR_A variants or empty vector after booster immunization (**Series II, Table 1**). Percent of responding CD8+ T cells was assessed by flow cytometry with ICCS (see Materials and Methods for details). Sequences of peptides used in stimulation are presented in Fig. 1C.

Peptide	Cytokine	Groups compared	Statistical test*	p value
A1-15	IFN-γ<br><br>Kruskal-Wallis<br>p=0.03	PR_Ai versus PR_Ai2mut	Mann-Whitney	0,5333
		PR_Ai3mut versus naive	Mann-Whitney	>0,9999
		(PR_Ai + PR_Ai2mut) versus (PR_Ai3mut + naïve)	Mann-Whitney	0,0095
	IL-2<br><br>Kruskal-Wallis<br>p=0.03	PR_Ai versus PR_Ai2mut	Mann-Whitney	0,5333
		PR_Ai3mut versus naive	Mann-Whitney	>0,9999
		(PR_Ai + PR_Ai2mut) versus (PR_Ai3mut + naïve)	Mann-Whitney	0,0095
	TNF-α<br><br>Kruskal-Wallis<br>p=0.04	PR_Ai versus PR_Ai2mut	Mann-Whitney	>0,9999
		PR_Ai3mut versus naive	Mann-Whitney	0,6667
		(PR_Ai + PR_Ai2mut) versus (PR_Ai3mut + naïve)	Mann-Whitney	0,0095
	IFN-γ/ IL-2/ TNF-α<br><br>Kruskal-Wallis<br>p=0.005	PR_Ai versus PR_Ai2mut	Mann-Whitney	0,1333
		PR_Ai3mut versus naive	Mann-Whitney	>0,9999
		(PR_Ai + PR_Ai2mut) versus (PR_Ai3mut + naïve)	Mann-Whitney	0,0095
AB71-85	IFN-γ	All groups	Kruskal-Wallis	0,9473
	IL-2	All groups	Kruskal-Wallis	0,3492
	TNF-α	All groups	Kruskal-Wallis	0,2800

	IFN-γ/ IL-2/ TNF-α<br><br>Kruskal-Wallis<br>p=0.04	PR_Ai / PR_Ai3mut / naive	Kruskal-Wallis	0,2095
		PR_Ai2mut versus (PR_Ai + PR_Ai3mut + naïve)	Mann-Whitney	0,0444
AB71-85dr	IFN-γ<br><br>Kruskal-Wallis<br>p=0.1	PR_Ai / PR_Ai2mut / naive	Kruskal-Wallis	0,6952
		PR_Ai3mut versus (PR_Ai + PR_Ai2mut + naïve)	Mann-Whitney	0,0444
	IL-2	All groups	Kruskal-Wallis	0,2203
	TNF-α	All groups	Kruskal-Wallis	0,3492
	IFN-γ/ IL-2/ TNF-α<br><br>Kruskal-Wallis<br>p=0.05	PR_Ai / PR_Ai2mut / naive	Kruskal-Wallis	0,2190
		PR_Ai3mut versus (PR_Ai + PR_Ai2mut + naïve)	Mann-Whitney	0,0444
AB75-84	IFN-γ<br><br>Kruskal-Wallis<br>p=0.02	PR_Ai versus PR_Ai2mut	Mann-Whitney	0,8000
		PR_Ai3mut versus naive	Mann-Whitney	0,3333
		(PR_Ai+PR_Ai2mut) versus (PR_Ai3mut + naïve)	Mann-Whitney	0,0095
	IL-2<br><br>Kruskal-Wallis<br>p=0.02	PR_Ai versus PR_Ai2mut	Mann-Whitney	0,5333
		PR_Ai3mut versus naive	Mann-Whitney	0,6667
		(PR_Ai+PR_Ai2mut) versus (PR_Ai3mut + naïve)	Mann-Whitney	0,0095
	TNF-α<br><br>Kruskal-Wallis<br>p=0.04	PR_Ai versus PR_Ai2mut	Mann-Whitney	0,5333
		PR_Ai3mut versus naive	Mann-Whitney	0,3333

		(PR_Ai+PR_Ai2mut) versus (PR_Ai3mut + naïve)	Mann-Whitney	0,0190
	IFN-γ/ IL-2/ TNF-α<br><br>Kruskal-Wallis p=0.0013	PR_Ai versus PR_Ai2mut	Mann-Whitney	0,1333
		PR_Ai3mut versus naive	Mann-Whitney	0,3333
		(PR_Ai+PR_Ai2mut) versus (PR_Ai3mut + naïve)	Mann-Whitney	0,0095
A76-90	IFN-γ	All groups	Kruskal-Wallis	0,1765
	IL-2	All groups	Kruskal-Wallis	0,0930
	TNF-α<br><br>Kruskal-Wallis p=0.04	PR_Ai versus PR_Ai2mut	Mann-Whitney	>0,9999
		PR_Ai3mut versus naive	Mann-Whitney	>0,9999
		(PR_Ai+PR_Ai2mut) versus (PR_Ai3mut + naïve)	Mann-Whitney	0,0095
	IFN-γ/ IL-2/ TNF-α<br><br>Kruskal-Wallis p=0.02	PR_Ai versus PR_Ai2mut	Mann-Whitney	0,8000
		PR_Ai3mut versus naive	Mann-Whitney	0,3333
		(PR_Ai+PR_Ai2mut) versus (PR_Ai3mut + naïve)	Mann-Whitney	0,0095
A76-90dr	IFN-γ	All groups	Kruskal-Wallis	0,5517
	IL-2	All groups	Kruskal-Wallis	0,1121
	TNF-α	PR_Ai3mut versus PR_Ai	Kruskal-Wallis with Dunn's multiple comparisons test	0,0228
		PR_Ai3mut versus PR_Ai2mut		0,7430
		PR_Ai3mut versus naive		>0,9999
	IFN-γ/ IL-2/ TNF-α	PR_Ai / PR_Ai2mut / naive	Kruskal-Wallis	0,6952

	Kruskal-Wallis p=0.16	PR_Ai3mut versus (PR_Ai + PR_Ai2mut + naïve)	Mann-Whitney	0,0444

* Responses to a peptide registered by the groups were first assessed by Kruskall-Wallis test. If p value for a selection of groups was >0.05, samples were assumed to belong to the same group, and groups were fused. Fused groups are listed in brackets. Two types of fusions were made: of (PR_Ai + PR_Ai2mut) as two immunogens inducing similar pattern of cellular immune response, and (PR_Ai3mut + naïve) as not exhibiting this pattern. The other was (PR_Ai + PR_Ai2mut + naïve) as not exhibiting the pattern of immune response characteristic to PR_Ai3mut. Values of p<0.05 in Kruskall-Wallis test indicated that entries belong to different groups. Significance of differences between the groups was further analyzed in pairs using Mann Whitney U-test.

Supplementary Table S4. Welfare chart for groups of BALB/c mice (n=6 per group) DNA immunized with PR_Ai, or equimolar mix of PR_Ai2mut and PR_AI3mut (DR PR mix), or empty vector pVAX1, or PBS via prime/boost regimen followed by electroporation (Table 1, Series III). General health condition of the animals was assessed by a veterinary doctor daily according to the chart A without scoring. An in-depth clinical examination was performed weekly according to the chart B with scoring. For each animal the general health condition was described by marking checkboxes corresponding to normal or altered condition. Altered conditions, if noted, were to be further specified in the “Comments” section. General condition of the animals corresponded to physiological norm throughout whole study period. Animals from all groups actively consumed feed and water. Social behavior, pose, coordination, breathing, defecation, urination, reaction to stimuli, muscle tone, the condition of the coat, mucous membranes, eyes, nasal cavity, oral cavity and other parameters corresponded to the physiological norm. The condition of all animals at all time-points was registered as normal. Electroporation caused round-shaped burns 3–4 mm in diameter on the skin of all animals, independently of the injected agent; burns resolved before the first weekly examination.

**A. Parameters assessed daily**	
Behavior	Normal
	Altered (depression/agitation)
Reaction to stimuli	Normal
	Altered (decrease/increase)
Skin	Normal
	Altered (redness/pallor/cyanosis/icteric)
Mucous membranes	Normal
	Altered (redness/pallor/cyanosis/icteric)
Discharge (nasal/eyes/anal/uretral)	Normal
	Altered
Muscle tone	Normal
	Altered (decrease/increase)
Coordination	Normal
	Altered (ataxia/hyperkinesis)
Breathing	Normal
	Altered (dyspnea/pathologic)
Feed and water consumption	Normal
	Altered (decrease/increase)
Death	

Parameters assessed weekly			
Parameters		Mark if <u>yes</u>	Comments
**Parameters assessed weekly in cage**			
Behavior	Normal	☐	
	Altered (depression/agitation)	☐	
Social interactions	Normal	☐	
	Altered (aggression)	☐	
**Parameters assessed weekly in hands**			
Reaction to stimuli	Normal	☐	
	Altered (decrease/increase)	☐	
Body composition	Normal	☐	
	Altered (wasting/obesity)	☐	
Muscle tone	Normal	☐	
	Altered (decrease/increase)	☐	
Fur	Normal	☐	
	Altered (disheveled/shedding/dull/dirty/discolored)	☐	
Skin	Normal	☐	
	Altered (describe: turgor, color, wounds, palpable mass)	☐	
Mucous membranes	Normal	☐	
	Altered (redness/pallor/cyanosis/icteric)	☐	
Eyes	Normal	☐	
	Altered (exophthalmos/wounds/redness/discharge)	☐	
Ears	Normal	☐	
	Altered (inflammation/discharge)	☐	
Nose	Normal	☐	

	Altered (serous discharge/purulent discharge/bloody discharge)	☐	
Mouth	Normal	☐	
	Altered (hypersalivation, blood, damaged teeth)	☐	
**Parameters assessed weekly in open field**			
Pose	Normal	☐	
	Altered (stereotypic movements, laying on side)	☐	
Coordination	Normal	☐	
	Altered (ataxia/hyperkinesis)	☐	
Breathing	Normal	☐	
	Altered (dyspnea/pathologic)	☐	
Urine	Normal	☐	
	Altered (discoloration)	☐	
Feces	Normal	☐	
	Altered (diarrhea/blood/discoloration)	☐	
Other	Describe	☐	

Supplementary Table S5. Body mass assessment of the groups of BALB/c mice (n=6 per group) DNA immunized with PR_Ai, or equimolar mix of PR_Ai2mut and PR_AI3mut, or empty vector pVAX1, or PBS via prime/boost regimen followed by electroporation on day 12 after booster immunization (Table 1, Series III). Statistical analysis of body mass data at each time point was done using fixed-effects model with Geisser-Greenhouse correction followed by Tukey's multiple comparisons. Differences were considered significant at P<0.05. There were no significant differences in the body mass between the recipients of any of the plasmids and the control group receiving PBS.

Days after first immunization	Study group			
	PR_Ai (n=6)	DR PR mix (n=6)	pVAX (n=5*)	PBS (n=6)
	Mean±SD (g)	Mean±SD (g)	Mean±SD (g)	Mean±SD (g)
0 (Day of Prime)	23.7±1.9	24.3±1.3	23.8±1.3	24.3±1.2
1	22.3±1.5	23.4±1.3	23±1	24±1.5
3	22.4±2	23.5±1.1	23±0.9	23.8±1.2
4	22.5±1.7	23.7±1.5	23.4±0.8	24.1±1.2
5	23±1.9	24.1±1.5	24.1±0.7	24.4±1.1
6	23.3±2.1	24.3±1.6	24.2±0.8	24.7±1.3
7	23.4±2.2	24.5±1.5	24.2±0.8	24.7±1.3
8	23.3±2.2	24.6±1.5	24.3±0.8	25.3±1.4
11	23.5±2.3	25.3±1.7	24.7±1.2	25.6±1.4
12	23.9±2.3	25.3±1.9	24.6±1.2	25.5±1.3
13	24.2±2.3	25.4±1.8	25.1±1.1	26±1.4
14	24.5±2.2	25.7±1.7	25.4±1.1	26.4±1.5
15	24.4±2.4	26.1±1.9	25.6±1.1	26.5±1.6
18	24.8±2.4	26.1±1.9	25.7±1.1	26.4±1.7
19	24.9±2.1	26.2±1.8	26±1.3	26.8±1.5
20	24±2.3	25.8±1.9	25.2±1.5	26.3±1.3
22	24.4±2.4	25.6±2.2	25±1.4	26±1.2

**25 (Day 1 after Boost)**	24.3±2.6	25.8±1.9	24.8±2	26.5±1.5
**26**	24.5±2.5	25.8±1.9	24.8±2.3	26.8±1.5
**27**	24.9±2.5	26.2±2	24.9±2.7	27±1.4
**28**	25.1±2.6	26.4±1.6	24.9±2.3	27.3±1.3
**29**	24.9±2.5	26.3±1.8	24.9±2.4	26.6±1.2
**36**	24.7±2.6	25.9±1.5	24.3±2.4	26.4±1.1

* One mouse in group pVAX1 died during the experiment, as it did not recover from anesthesia after the first immunization.

Supplementary Table S6. Results of the complete blood count analysis for BALB/c mice (n=6 per group) DNA immunized with PR_Ai, or equimolar mix of PR_Ai2mut and PR_AI3mut (DR PR mix), or empty vector pVAX1, or PBS via prime/boost regimen followed by electroporation on day 12 after booster immunization (end-point of the study; **Table 1, Series III).** Data is presented as mean ± SD. Statistically significance of differences was assessed using Kruskal-Wallis test with Dunn's multiple comparisons.

Immunogen	PR_Ai*		PR DR mix*		PR_Ai**		PR DR mix**		pVAX1**		PBS	
Assessment day	day 1	day 12	day 1	day 12	day 1	day 12	day 1	day 12	day 1	day 12	day 1	day 12
unclassified leukocytes (MID)	2.47+0.33	2.90+0.30	2.37+0.31	2.37+0.22	2.47+0.33	2.90+0.30	2.37+0.31	2.37+0.2 2	2.72+0.48	2.46+0.34	2.72+0.22	2.73+0.67
unclassified leukocytes (MID), Stat***	ns	ns	ns	ns	ns	ns	ns	ns	ns	ns	N/A	N/A

-* compared to pVAX1

-** compared to PBS

- ***Statistic was done with Kruskal-Wallis test with Dunn's multiple comparisons correction

Supplementary Table S7. Bone marrow composition of BALB/c mice (n=6 per group) DNA immunized with PR_Ai, or equimolar mix of PR_Ai2mut and PR_AI3mut (DR PR mix), or empty vector pVAX1, or PBS via prime/boost regimen followed by electroporation on day 12 after booster immunization (end-point of the study; **Table 1, Series III).** Statistically significance of differences were assessed using Kruskal-Wallis test with Dunn's multiple comparisons.

Immunogen	PR_Ai*		PR DR mix*		PR_Ai**		PR DR mix**		pVAX1**		PBS	
Assessment day	day 1	day 12	day 1	day 12	day 1	day 12	day 1	day 12	day 1	day 12	day 1	day 12
Erythroblasts	0,33+0,52	0,33+0,52	0,50+0,55	0,50+0,55	0,33+0,52	0,33+0,52	0,50+0,55	0,50+0,55	0,33+0,52	0,20+0,45	0,50+0,55	0,33+0,52
Erythroblasts, Stat***	ns	ns	ns	ns	ns	ns	ns	ns	ns	ns	N/A	N/A
Proerythroblasts	0,33+0,52	0,33+0,52	0,33+0,52	0,33+0,52	0,33+0,52	0,33+0,52	0,33+0,52	0,33+0,52	0,33+0,52	0,40+0,55	0,33+0,52	0,50+0,55
Proerythroblasts, Stat***	ns	ns	ns	ns	ns	ns	ns	ns	ns	ns	N/A	N/A
Basophylic erythroblasts	3,67+0,52	4.33+1.03	4.33+1.03	3.83+1.17	3,67+0,52	4.33+1.03	4.33+1.03	3.83+1.17	3.83+0.75	3.60+0.55	4.17+0.75	4.00+0.63
Basophylic erythroblasts, Stat***	ns	ns	ns	ns	ns	ns	ns	ns	ns	ns	N/A	N/A
Polychromato-philic erythroblasts	11,50+1,05	12.83+1.17	12.67+1.21	13.17+1.72	11,50+1,05	12.83+1.17	12.67+1.21	13.17+1.72	12.00+0.63	12.80+0.84	12.50+1.87	13.00+1.55
Polychromato-philic erythroblasts, Stat***	ns	ns	ns	ns	ns	ns	ns	ns	ns	ns	N/A	N/A
Oxyphilic erythroblasts	14,33+0,82	15.33+1.51	13,67+0,82	14.83+1.47	14,33+0,82	15.33+1.51	13,67+0,82	14.83+1.47	14,00+1,67	14.60+1.14	13,67+2,42	15.00+1.26
Oxyphilic erythroblasts, Stat***	ns	ns	ns	ns	ns	ns	ns	ns	ns	ns	N/A	N/A

Lymphocytes	17.83+1.17	19.00+1.55	15.83+1.17	18.50+1.38	17.83+1.17	19.00+1.55	15.83+1.17	18.50+1.38	16.17+1.83	18.40+1.34	16.67+1.75	18.50+1.64
Lymphocytes, Stat***	ns	ns	ns	ns	ns	ns	ns	ns	ns	ns	N/A	N/A
Monocytes	8.00+1.26	7.17+0.98	7.33+0.82	7.17+0.75	8.00+1.26	7.17+0.98	7.33+0.82	7.17+0.75	7.33+0.82	7.40+0.55	7.67+1.03	7.33+0.82
Monocytes, Stat***	ns	ns	ns	ns	ns	ns	ns	ns	ns	ns	N/A	N/A
Reticuloendothe-lial cells	1.50+0.55	0.83+0.41	1.33+0.52	1.17+0.41	1.50+0.55	0.83+0.41	1.33+0.52	1.17+0.41	1.50+0.55	1.20+0.45	1.50+0.55	1.17+0.41
Reticuloendothe-lial cells, Stat***	ns	ns	ns	ns	ns	ns	ns	ns	ns	ns	N/A	N/A
Myeloblasts	0.67+0.52	0.67+0.52	0.67+0.52	0.50+0.55	0.67+0.52	0.67+0.52	0.67+0.52	0.50+0.55	0.83+0.41	0.60+0.55	0.83+0.41	0.50+0.55
Myeloblasts, Stat***	ns	ns	ns	ns	ns	ns	ns	ns	ns	ns	N/A	N/A
Promyelocytes	1.00+0.00	1.00+0.89	0.67+0.52	1.117+0.75	1.00+0.00	1.00+0.89	0.67+0.52	1.117+0.75	0.83+0.75	0.80+0.45	0.83+0.75	0.83+0.41
Promyelocytes, stat***	ns	ns	ns	ns	ns	ns	ns	ns	ns	ns	N/A	N/A
Myelocytes	5.00+0.41	4.00+1.26	5.17+0.41	3.83+0.75	5.00+0.41	4.00+1.26	5.17+0.41	3.83+0.75	5.50+0.55	3.80+0.84	5.67+0.52	4.00+0.89
Myelocytes, Stat***	ns	ns	ns	ns	ns	ns	ns	ns	ns	ns	N/A	N/A
Band neutrophils	7.00+1.26	8.33+1.03	7.83+0.75	1.83+1.83	7.00+1.26	8.33+1.03	7.83+0.75	1.83+1.83	8.17+0.75	8.40+0.55	7.50+0.55	8.83+0.75
Band neutrophils, Stat***	ns	ns	ns	ns	ns	ns	ns	ns	ns	ns	N/A	N/A
Segmented neutrophils	22,83+1,33	19,33+2,07	23.00+1.26	19,83+0,75	22,83+1,33	19,33+2,07	23.00+1.26	19,83+0,75	22.33+2.50	21,60+1,95	21,67+1,51	19,67+2,25
Segmented neutrophils, Stat***	ns	ns	ns	ns	ns	ns	ns	ns	ns	ns	N/A	N/A
Basophils	0.17+0.41	0.67+0.52	0.50+0.55	0.50+0.55	0.17+0.41	0.67+0.52	0.50+0.55	0.50+0.55	0.33+0.52	0.40+0.55	0.33+0.52	0.50+0.55
Basophils, Stat***	ns	ns	ns	ns	ns	ns	ns	ns	ns	ns	N/A	N/A

Eosinophils	1.33+0.52	1.33+0.82	1.00+0.00	1.00+0.00	1.33+0.52	1.33+0.82	1.00+0.00	1.00+0.00	1.17+0.41	1.40+0.55	1.33+0.52	1.17+0.41
Eosinophils, Stat***	ns	ns	ns	ns	ns	ns	ns	ns	ns	ns	N/A	N/A
Other cells	1.17+0.41	0.50+0.55	1.00+0.00	0.83+0.41	1.17+0.41	0.50+0.55	1.00+0.00	0.83+0.41	0.67+0.52	0.80+0.45	1.00+0.00	0.83+0.41
Other cells, Stat***	ns	ns	ns	ns	ns	ns	ns	ns	ns	ns	N/A	N/A

-* compared to pVAX1

-** compared to PBS

- ***Statistic was done with Kruskal-Wallis test with Dunn’s multiple comparisons correction

Supplementary Table S8. Mass of axillary and inguinal lymph nodes of BALB/c mice (n=6 per group) DNA immunized with PR_Ai, or equimolar mix of PR_Ai2mut and PR_AI3mut (DR PR mix), or empty vector pVAX1, or PBS via prime/boost regimen followed by electroporation on day 12 after booster immunization (end-point of the study; **Table 1, Series III).** Data represent organ mass as % to body mass, mean±SD. Statistical significance of the differences was assessed by the Kruskal-Wallis test with Dunn's multiple comparisons test.

Immunogen	PR_Ai*		PR DR mix*		PR_Ai**		PR DR mix**		pVAX1**		PBS	
Assessment day	day 1	day 12	day 1	day 12	day 1	day 12	day 1	day 12	day 1	day 12	day 1	day 12
Inguinal lymph node	0.03+0.01	0.03+0.01	0.02+0.01	0.03+0.01	0.03+0.01	0.03+0.01	0.02+0.01	0.03+0.01	0.03+0.01	0.04+0.01	0.02+0.01	0.12+0.25
Inguinal lymph node, Stat***	ns	ns	ns	ns	ns	ns	ns	ns	ns	ns	N/A	N/A

-* compared to pVAX1

-** compared to PBS

- ***Statistic was done with Kruskal-Wallis test with Dunn's multiple comparisons correction

Table S9. Results of the analysis of blood biochemistry of BALB/c mice DNA immunized with PR_Ai, or equimolar mix of PR_Ai2mut and PR_AI3mut (DR PR mix), or empty vector pVAX1, or PBS via prime/boost regimen followed by electroporation on day 12 after booster immunization (end-point of the study; **Table 1, Series III).** Biochemical analysis including measurement of the levels of alanine aminotransferase (ALT), aspartate aminotransferase (AST), alkaline phosphatase (ALP), lactate dehydrogenase (LDH), total protein (TP), albumin (ALB), urea (UREA), glucose (GLC), cholesterol (CHOL), triglycerides (TG), sodium (Na) and potassium (K) was performed using Stat Fax 4500+ biochemical analyzer (Awareness technology, Ramsey, MN, USA) according to manufacturer's protocols. Values represent mean ± SD. Statistical significance of the differences was assessed by the Kruskal-Wallis test with Dunn's multiple comparisons test. Differences were considered significant at P<0.05.

Immunogen	PR_Ai*		PR DR mix*		PR_Ai**		PR DR mix**		pVAX1**		PBS	
Assessment day	day 1	day 12	day 1	day 12	day 1	day 12	day 1	day 12	day 1	day 12	day 1	day 12
AST, U/L	286,92+52,27	230,34+35,46	335,82+105,07	190,38+70,20	286,92+52,27	230,34+35,46	335,82+105,07	190,38+70,20	342,18+87,25	324,54+115,57	313,38+49,13	290,28+71,14
AST, Stat***	ns	ns	ns	ns	ns	ns	ns	ns	ns	ns	N/A	N/A
ALP, U/L	306,80+38,43	428,60+101,75	346,20+6,61	315,80+71,45	306,80+38,43	428,60+101,75	346,20+6,61	315,80+71,45	309,20+44,43	367,00+146,66	323,00+93,46	402,80+112,88
ALP, Stat***	ns	ns	ns	ns	ns	ns	ns	ns	ns	ns	N/A	N/A
LDH, U/L	1992,20+486,92	2481,00+598,30	2152,00+623,69	2181,60+660,74	1992,20+486,92	2481,00+598,30	2152,00+623,69	2181,60+660,74	2449,20+478,52	2931,70+416,91	2095,80+175,76	2726,40+460,58
LDH, Stat***	ns	ns	ns	ns	ns	ns	ns	ns	ns	ns	N/A	N/A
Albumin, g/L	36,60+11,24	43,96+2,11	40,00+2,35	42,62+7,32	36,60+11,24	43,96+2,11	40,00+2,35	42,62+7,32	41,40+4,56	44,44+7,71	36,40+2,51	42,04+7,06
Albumin, Stat***	ns	ns	ns	ns	ns	ns	ns	ns	ns	ns	N/A	N/A
Glucose, mmol/L	8,45+2,41	7,89+1,43	8,57+2,16	6,43+1,20	8,45+2,41	7,89+1,43	8,57+2,16	6,43+1,20	7,20+1,13	7,08+3,17	10,06+1,33	6,85+1,37
Glucose, Stat***	ns	ns	ns	ns	ns	ns	ns	ns	ns	ns	N/A	N/A
Na, mmol/L	121,60+5,18	127,46+9,67	120,20+11,19	124,74+4,57	121,60+5,18	127,46+9,67	120,20+11,19	124,74+4,57	121,80+7,16	121,78+6,03	119,80+4,09	124,10+3,94

Na, Stat***	ns	ns	ns	ns	ns	ns	ns	ns	ns	ns	N/A	N/A
K, mmol/L	7,66+1,31	7,24+1,52	8,92+1,53	6,50+2,49	7,66+1,31	7,24+1,52	8,92+1,53	6,50+2,49	8,68+1,05	7,54+2,32	7,12+1,75	8,16+1,18
K, Stat***	ns	ns	ns	ns	ns	ns	ns	ns	ns	ns	N/A	N/A

-* compared to pVAX1

-** compared to PBS

- ***Statistic was done with Kruskal-Wallis test with Dunn’s multiple comparisons correction

Supplementary Table S10. Correlation of the biochemical parameters of the blood of BALB/c mice DNA immunized with PR_Ai, or equimolar mix of PR_Ai2mut and PR_AI3mut (DR PR mix), or empty vector pVAX1, or PBS via prime/boost regimen with electroporation on day 12 after booster immunization (Table 1, Series III). Parameters, mean per group with SD, are presented in **Table 3** and **Suppl Table S9**. Correlations were analysed using nonparametric Spearman Rank Correlation test (Statistica 11, Tibco, USA). P values <0.05 were considered significant.

Biochemical blood parameters	Correlated to	r value in Spearman test	p	r^2
Triglycerides (TGC)	Urea	0.4279	0.00590	0.1831
	Total protein	0.0044	0.97860	0.0000
	ALB	0.3148	0.04790	0.0991
	Glucose	0.0779	0.63300	0.0061
	Cholesterole	0.0527	0.74680	0.0028
Urea	Total protein	-0.0519	0.75030	0.0027
	ALB	0.2833	0.07650	0.0803
	Glucose	-0.0796	0.62520	0.0063
	Cholesterole	0.0634	0.69760	0.0040
	TGC	0.4279	0.00590	0.1831
Cholesterole	Total protein	0.6144	0.00002	0.3775
	ALB	0.6535	0.00000	0.4271
	Urea	0.0634	0.69760	0.0040
	Glucose	0.0596	0.71470	0.0036
	TGC	0.0527	0.74680	0.0028
Total protein	ALB	0.4095	0.00870	0.1677
	Urea	-0.0519	0.75030	0.0027

	Glucose	0.0210	0.89760	0.0004
	Cholesterole	0.6144	0.00002	0.3775
	TGC	-0.0044	0.97860	0.0000
Albumine (ALB)	Total protein	0.4095	0.00870	0.1677
	Urea	0.2833	0.07650	0.0803
	Glucose	0.0592	0.71650	0.0035
	Cholesterole	0.6535	0.00000	0.4271
	TGC	0.3148	0.04790	0.0991
Glucose	Total protein	0.0210	0.89760	0.0004
	ALB	0.0592	0.71650	0.0035
	Urea	-0.0796	0.62520	0.0063
	Cholesterole	0.0596	0.71470	0.0036
	TGC	-0.0779	0.63300	0.0061

* One mouse in group pVAX1 died during the experiment, as it did not recover from anesthesia after the first immunization, resepctive measurements are missing.

Supplementary Figures

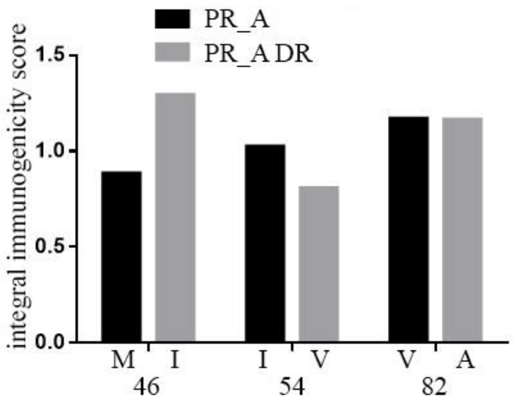

Supplementary Figure S1. Comparison of the integral immunogenicity scores predicting MHC class I immunogenicity of the regions of the consensus HIV-1 FSU_A protease (PR_A) harbouring drug resistance (DR) mutations 46I, 54V, and 82A. Respective regions from PR_A are dubbed "PR_A wild-type", and from harbouring DR mutation, PR_A DR. Integral immunogenicity score is the sum of the prediction scores of four peptides derived from respective region which have the highest scores of predicted MHC class I immunogenicity according to IEDB Immunogenicity prediction tool (see Materials and Methods for details).

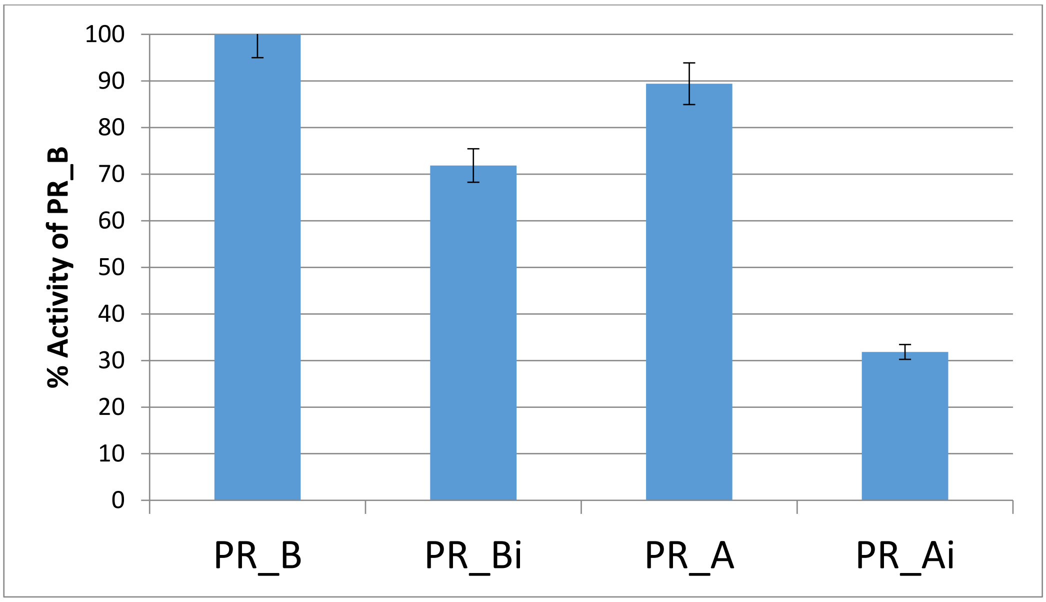

Supplementary Figure S2. Enzymatic activity of the variants of HIV-1 protease: consensus clade A FSU_A protease(PR_A), PR_A with inactivation mutation D25N (PR_Ai), clade B HXB-2 (PR_B) and PR_B with inactivation mutation D25N (PR_Bi). . Protease activity was measured in the lysates of HeLa cells transfected with eukaryotic expression vectors encoding respective PR variants (Table 1). Approximately 400 000 cells were transfected with pVAX1_PR_B, pVAX1_PR_Bi, pVAX1_PR_A, pVAX1_PR_Ai (Suppl. Table 1) with the efficacy of 65%, 48 hours post transfection cells were lysed and assessed for protease activity using FRET assay (SensoLyte 490, Anaspec, CA, USA) as described by the manufacturer. Activity was normalized to the activity of PR_B. Data represent mean of at least two measurements, with standard error -f we can call 5% deviation a standard error?? Otherwise, add SD.The actual loss of enzymatic activity after introduction of D25N could have been higher, if PR activity was normalized to the intracellular level of the protein in expressing cells, high for inactive and low for active PR variants (Fig. 3) due to autocleavage (Mildner et al., 1994). This, however, could not be done since such measurement relied on treatment of the cells with protease inhibitor cocktail to prevent enzyme autocleavage, the cocktail would then interfere with the assessment of protease activity.

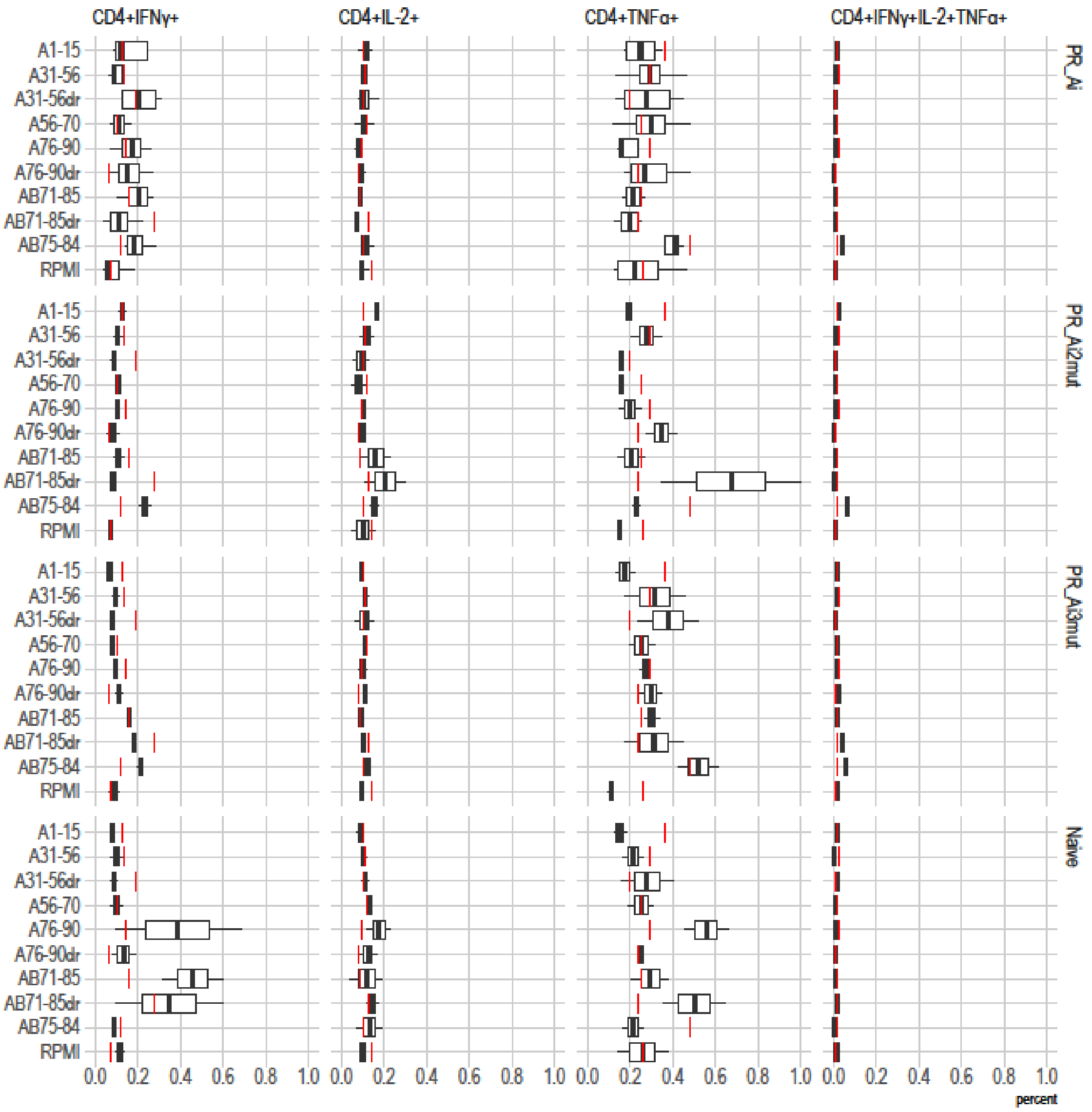

Supplementary Figure S3. CD4+ T cells of mice DNA immunized with PR_A variants exhibited no PR specific response to *in vitro* stimulation with peptides representing immunodominant epitopes of PR. BALB/c mice were immunized with plasmids encoding PR_Ai, or PR_Ai2mut, or PR_Ai3mut or empty vector pVAX1 (Table 1, Series II). Two weeks post booster immunization mice (n=5 per group) were sacrificed, their splenocytes were purified and frozen at -80° C. Week later, cells were thawed and subjected to stimulation with PR-derived peptides (Fig. 1C). IFN-γ, IL-2 and TNF-α and triple cytokine production by stimulated T cells was assessed by flow cytometry with ICCS as described in the Materials and Methods. Frequencies of cytokine positive CD4+ T-cells secreting IFN-γ, or IL-2, or TNF-α and triple IFN-γ/IL-2/TNF-α cytokine secreting cells illustrated by box with whiskers; red line illustrate data for pVAX1 immunized mice. CD4+ reactivity to PR-derived peptides in mice DNA immunized with PR_A variants did not differ from that exhibited by CD4+ T cells of naïve mice.

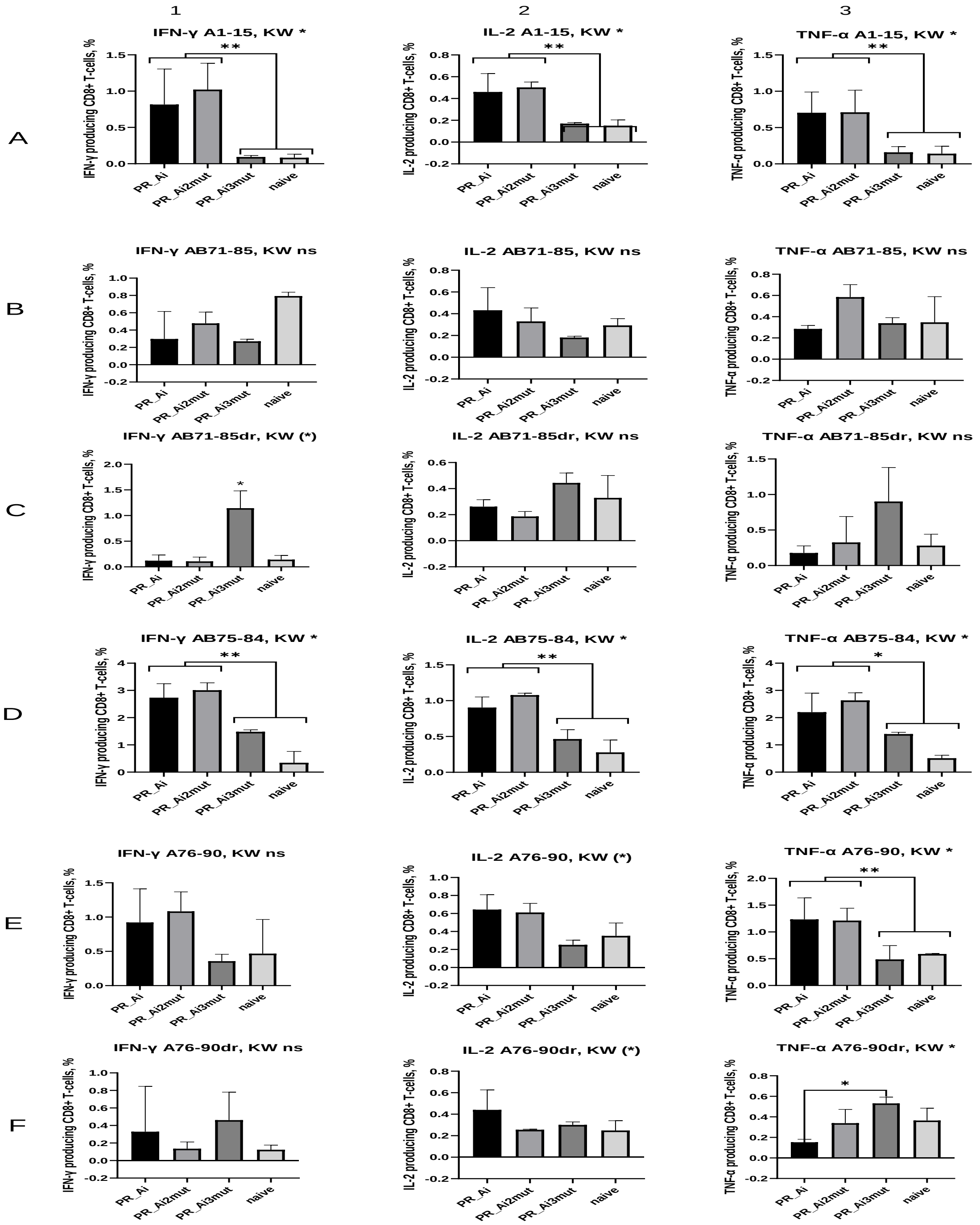

Supplementary Figure S4 CD8+ T cell response of mice DNA immunized with PR_A variants (Table 1, Series II) to stimulation with peptides representing immunodominant epitopes of PR. Percent of CD8+ T cells responding to stimulation with peptides A1-15 (A), A71-85 (B), A71-85dr with mutation V82A (C), AB75-84 (D), A76-90 (E) and A7690dr with V82A mutation (F) by production of IFN-γ (A-F, panel 1), IL-2 (A-F, panel 2), or TNF-α (A-F, panel 3). BALB/c mice were immunized with plasmids encoding PR_Ai, or PR_Ai2mut, or PR_Ai3mut or empty vector pVAX1 (Table 1, Series II). Two weeks post booster immunization mice (n=5 per group) were sacrificed, their splenocytes were purified and frozen at -80 °C. Week after, cells were thawed and subjected to stimulation with PR-derived peptides (Fig. 1C). T cell reactivity was assessed by flow cytometry with intracellular cytokine staining (ISSC) as described in the Materials and Methods. Difference between groups was first analyzed by Kruskal-Wallis, groups exhibiting no difference were pooled, and groups/pooled groups were re-analyzed by Mann Whitney U-test (as indicated in **Suppl Table S3**). KW*, $p<0.05$; KW (*), $p<0.1$; MW: *$p<0.05$, ** $p<0.01$.

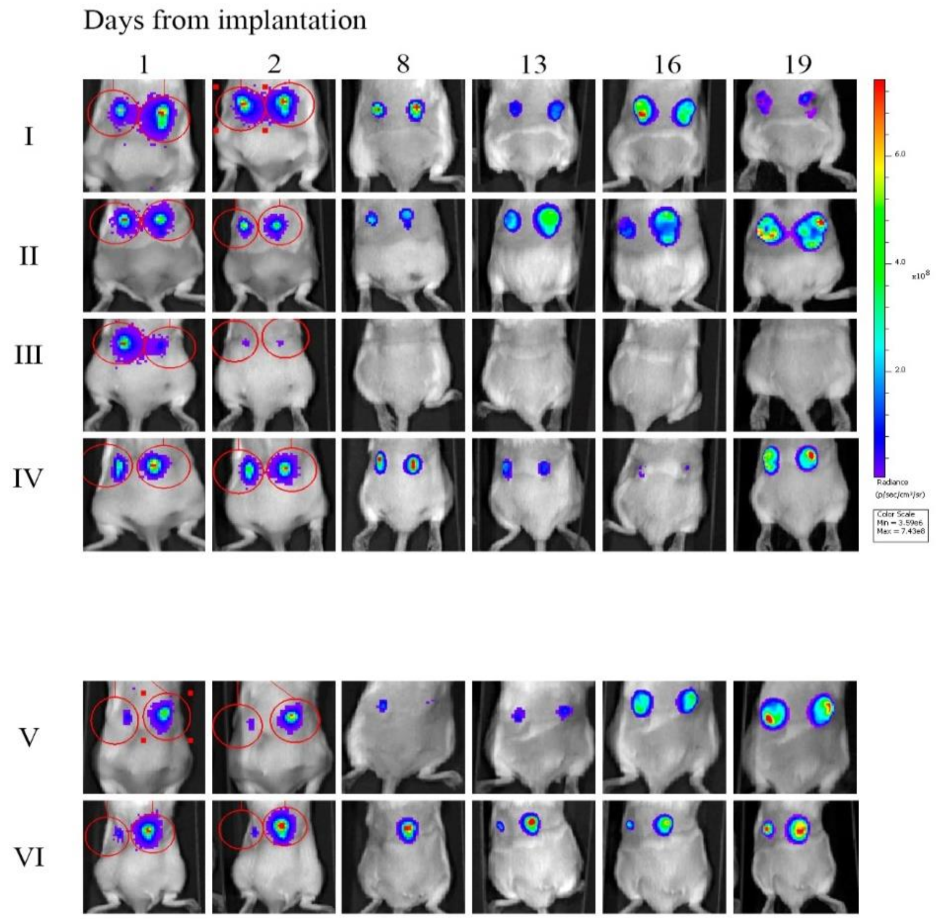

Supplementary Figure S5. Immunization with PR_Ai3mut identical to PR variant expressed by tumor cells protects mice against growth of PR_Ai3mut expressing adenocarcinoma cells, while no protection is rendered by immunization with PR variants different in three (PR_Ai) or even one amino acid residue (PR_Ai2mut). Mice were DNA immunized with PR_Ai (panel I), PR_Ai2mut (II); PR_Ai3mut (III) and challenged with 4T1luc2 cells expressing PR_Ai3mut (PR20.20), or with empty vector pVAX (panle IV) or PBS (V) and challenged with parental 4T1luc2 cells. Experimental scheme is presented in **Table 1, Series IV**. Growth of tumor cells at the sites of implantation was assessed by bioluminescent imaging (BLI, Spectrum, Perkin Elmer) on days 1, 2, 8, 13, 16 and 19 as depicted over the images. Panels represent dynamics of tumor growth in representative mice from each of the groups.

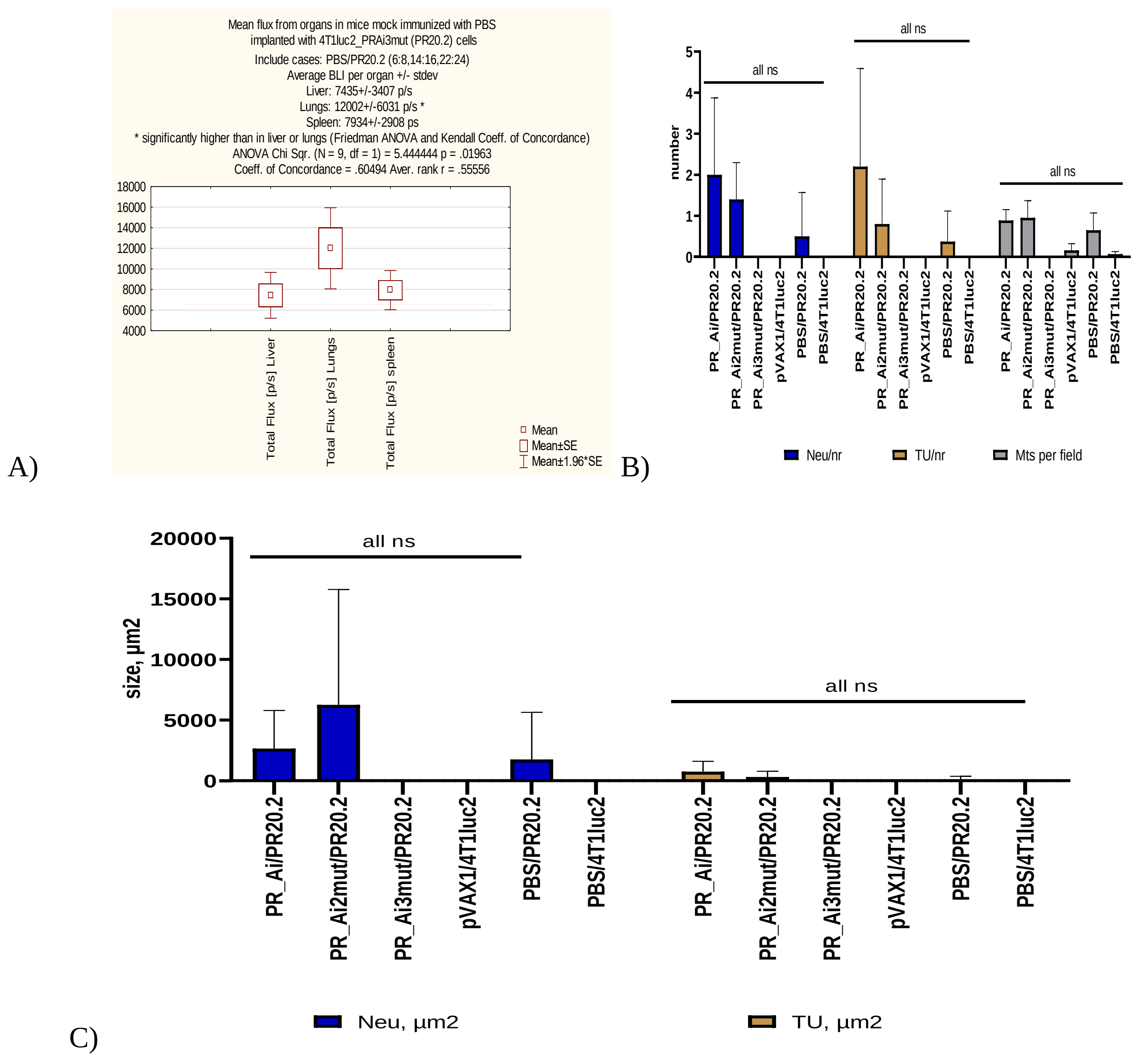

Supplementary Figure S6. Migrational and metastatic activity of murine adenocarcinoma 4T1luc2 cells expressing inactivated consensus HIV-1 FSU_A protease with drug resistance mutations M46I/I54V/V82A (PR20.2) and parental 4T1luc2 cells in mice DNA immunized with PR_Ai variants and control animals (Table 1, Series IV). Migration of PR20.2 cells into distal organs in control mice assessed by ex vivo organ imaging (A); Infiltration of PR20.2 and parental cells into the liver of mice DNA immunized with PR_Ai variants and control animals: total nn of neutrophils in all assessed sections (Neu, nn), total nn of tumor cells without immune infiltrates in all assessed sections (TU, nn), average nn of metastases per field in all assessed sections (Mts, nn) (B); Average size of all detected neutrophil infiltrates (Neu, μm^2), average size of metastasis formed by adenocarcinoma cells with no detected neutrophil infiltrates (TU, μm^2) (C). Assessed groups: mice DNA immunized with PR_Ai variants challenged with 4T1luc2_PR20.2 cells (n=5 for each immunogen), PBS immunized mice challenged with 4T1luc2_PR20.2 cells (n=9), vector immunized mice challenged with 4T1luc2 cells (n=5), PBS immunized controls challenged with 4T1luc2 cells. Histochemical assessment was done by independent pathologist on coded samples using computer-assisted morphometry with specialized NIS-Elements software (Nikon, Tokyo, Japan). Difference between the groups was analyzed by ordinary two-way ANOVA with Dunnet's multiple comparisons correction; ns - p>0.05.

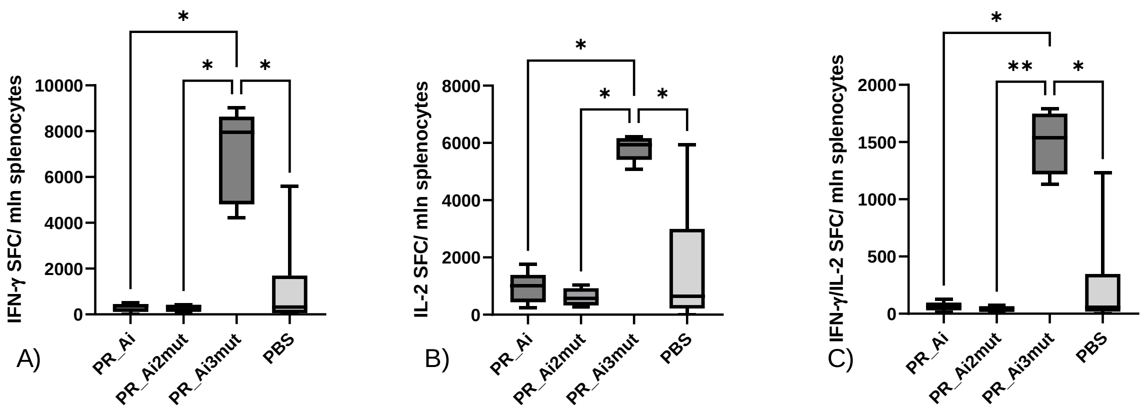

Supplementary Figure S7. Spleenocytes of mice DNA immunized with PR_Ai3mut demonstrate preserved capacity to react to stimulation with mitogen Conconavalin A (ConA) with production of IFN-γ (A), IL-2 (B) and dual production of IFN- γ /IL-2 (C), whereas this capacity is lost in mice DNA immunized with PR_A variants different from the one expressed by tumor cells (PR_Ai, PR_AI2mut). Mice were immunized as depicted in Series IV, Table 1, and post booster implanted with 4T1luc2_PR20.2 cells. Twenty-one days after challenge splenocytes of mice were stimulated in vitro with ConA for 18 h, as described in Materials and Methods. In vitro secretion of IFN-γ, IL-2, and dual secretion of IFN-γ/IL-2 was measured as the number of signal-forming units (sfu) per mln splenocytes, average per group. Differences were analyzed by Kruskal-Wallis with Dunn's multiple comparisons correction. *p<0.005, **p<0.01.

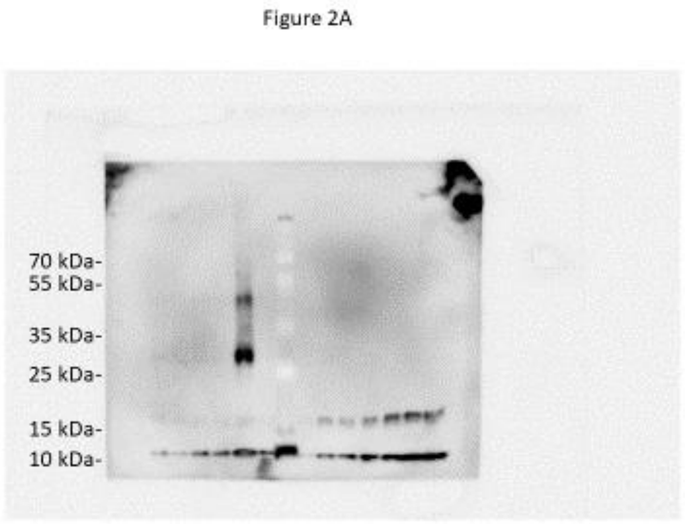

Supplementary Figure S8 Original Western Blot for Figure 2A

Figure 2B

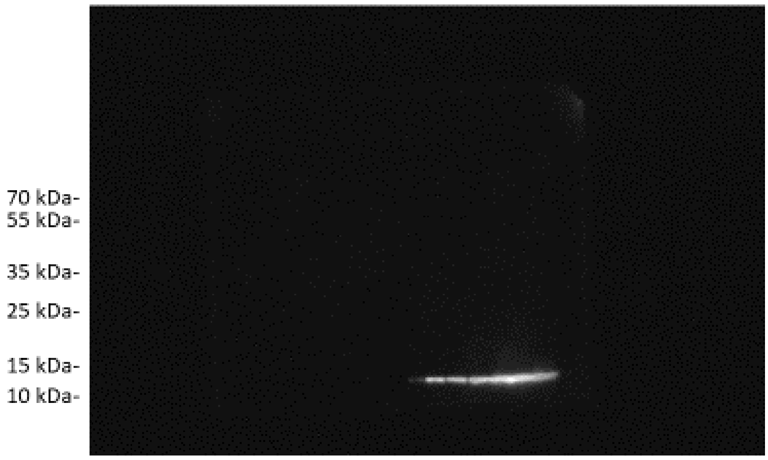

Supplementary Figure S9 Original Western Blot for Figure 2B

Figure 2C

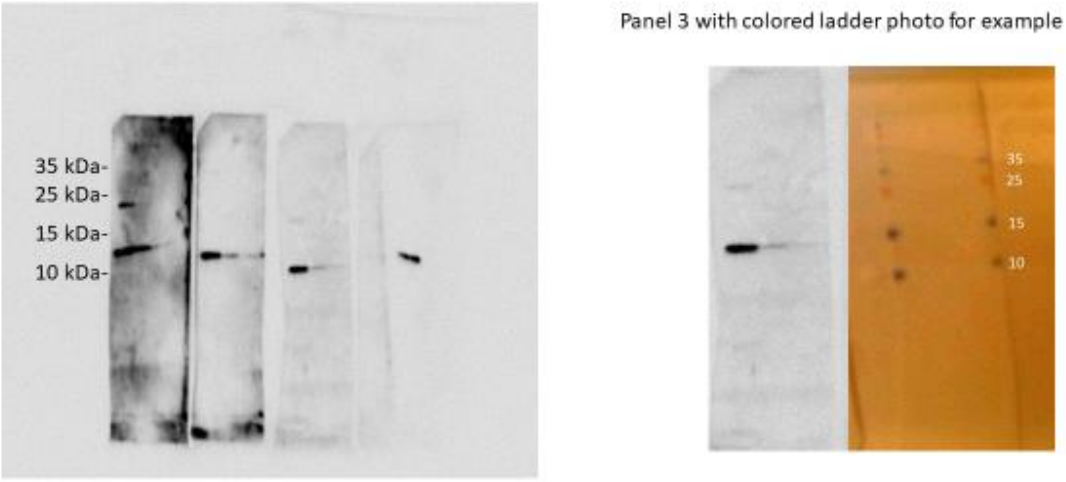

Supplementary Figure S10 Original Western Blot for Figure 2C

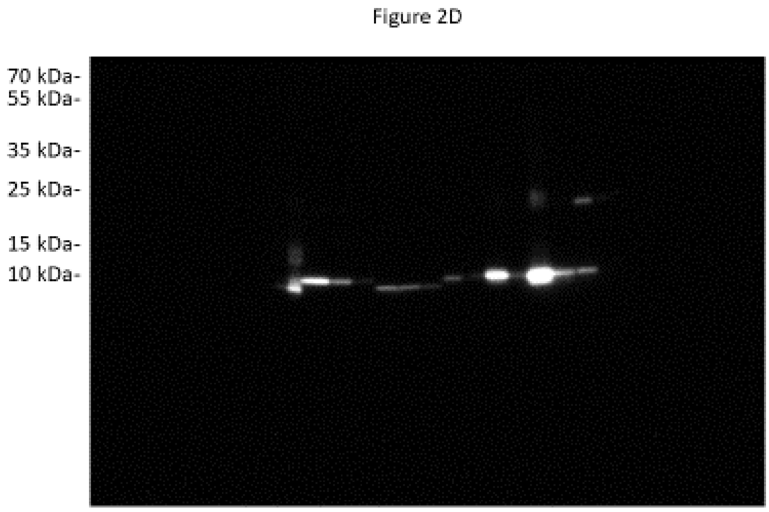

Supplementary Figure S11 Original Western Blot for Figure 2D

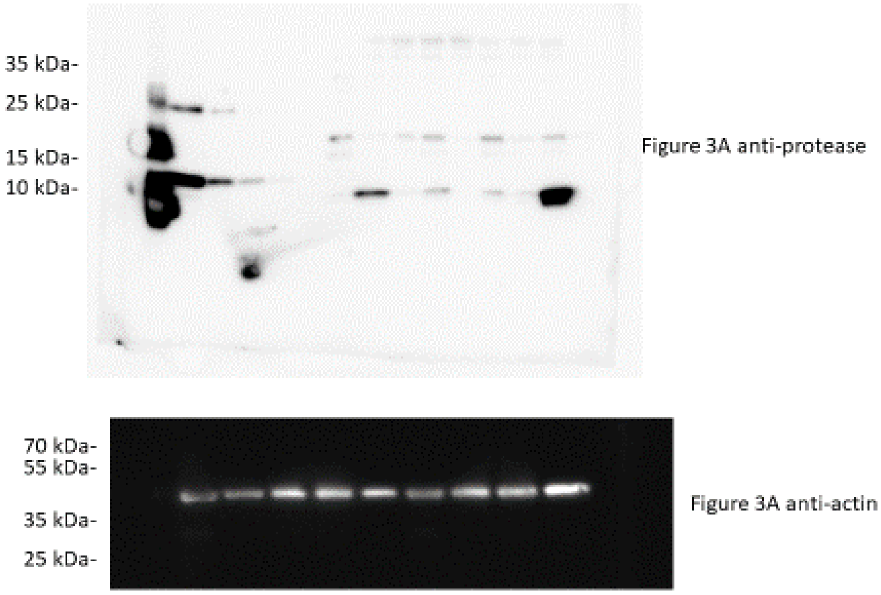

Supplementary Figure S12 Original Western Blot for Figure 3A

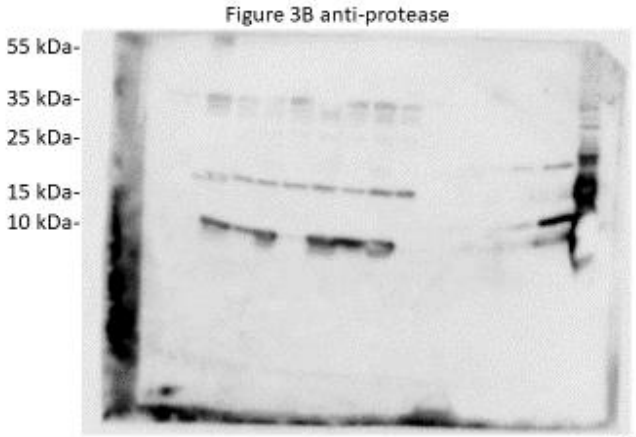

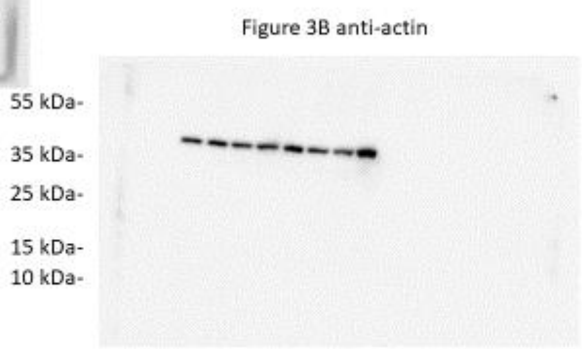

Supplementary Figure S13 Original Western Blot for Figure 3B
